# Supplementary material for: Recovery of tropical marine benthos after a trawl ban demonstrates linkage between abiotic and biotic changes
Source: Commun Biol. 2021 Feb 16;4:212. doi: 10.1038/s42003-021-01732-y (PMC7887210; doi:10.1038/s42003-021-01732-y)
Supplement: Supplementary file 2 — Supplementary Information [file 42003_2021_1732_MOESM2_ESM.pdf]

Supplementary Information

Recovery of tropical marine benthos after a trawl ban demonstrates linkage between abiotic and biotic changes

Zhi Wang[1,2,3], Kenneth M.Y. Leung[4,5,6*], Yik Hei Sung[7], David Dudgeon[4], Jian-Wen Qiu[1,2*]

[1] Department of Biology, Hong Kong Baptist University, Kowloon Tong, Kowloon, Hong Kong, China

[2] Southern Marine Science and Engineering Guangdong Laboratory, Guangzhou, China

[3] State Key Laboratory of Marine Environmental Science, College of Ocean and Earth Sciences, Xiamen University, Xiamen, 361102, China

[4] Division of Ecology and Biodiversity, School of Biological Sciences, The University of Hong Kong, Pokfulam, Hong Kong, China

[5] The Swire Institute of Marine Science, The University of Hong Kong, Cape D'Aguilar, Shek O, Hong Kong, China

[6] Present Address: State Key Laboratory of Marine Pollution and Department of Chemistry, City University of Hong Kong, Tat Chee Avenue, Kowloon, Hong Kong, China

[7] Science Unit, Lingnan University, Tuen Mun, Hong Kong, China

---

*Corresponding authors: kmyleung@cityu.edu.hk (K.M.Y. Leung) or qiujw@hkbu.edu.hk (J.W. Qiu)

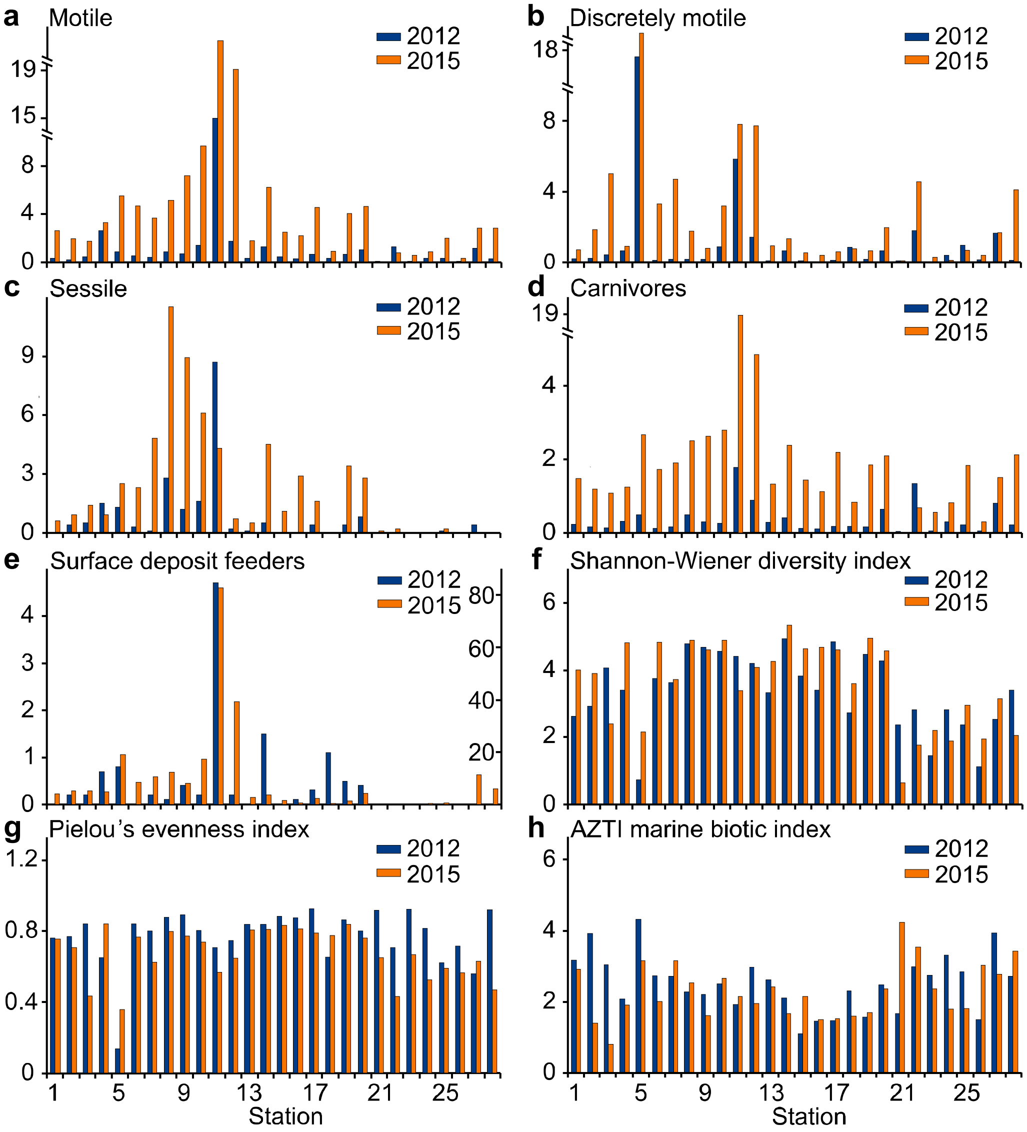

Supplementary Figure 1 Comparison of motility patterns, feeding modes and biotic indices across Hong Kong waters between the 2012 and 2015 surveys. a Abundance of motile benthos. **b** Abundance of discretely motile benthos. **c** Abundance of sessile benthos. **d** Abundance of carnivores. **e** Abundance of surface deposit feeders, with primary axis for the 2012 data and secondary axis for the 2015 data. **f** Shannon-Wiener diversity index (*H'*). **g** Pielou's evenness index (*J*). **h** AZTI Marine Biotic Index (AMBI). The biotic variables in each site in the diagrams are calculated from the pooled samples of the area of 0.5 m^2.

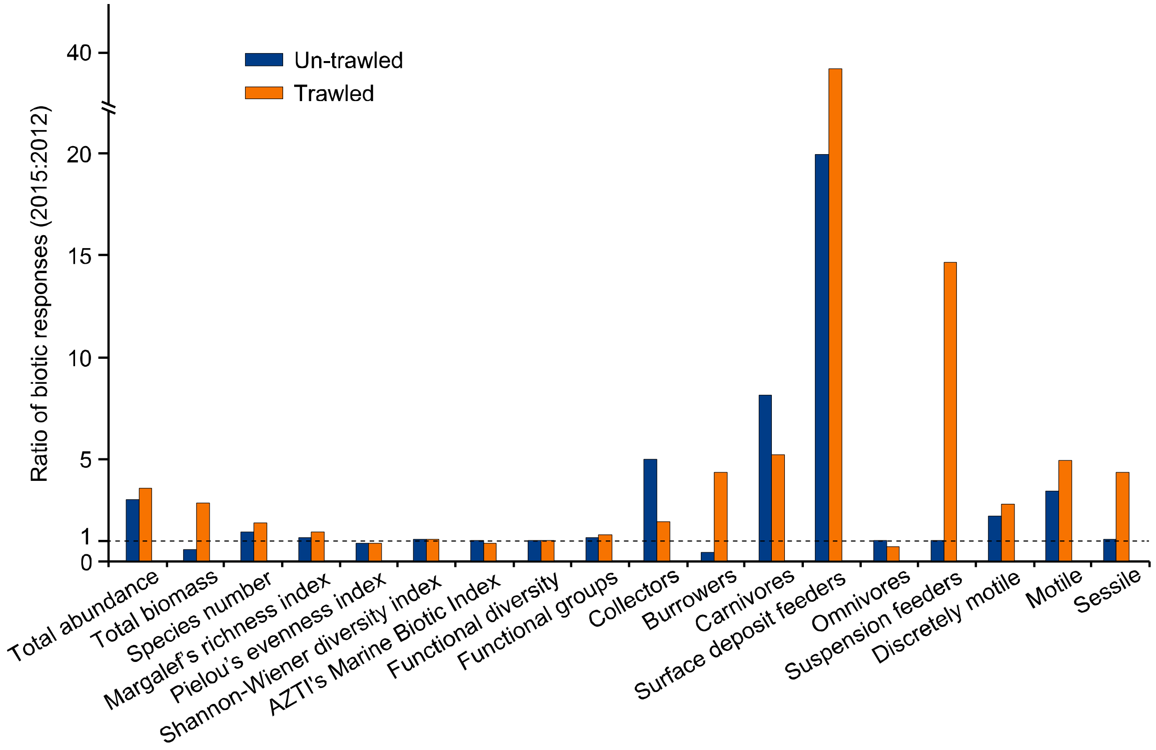

Supplementary Figure 2 Ratios of 18 biotic responses (2015:2012) in the surveyed sites. The histograms in blue represents data of un-trawled sites; and the histogram in orange represents data of trawled sites. The un-trawled and trawled sites refer to Supplementary Table 4. The dashed line represents the ratio of 1.

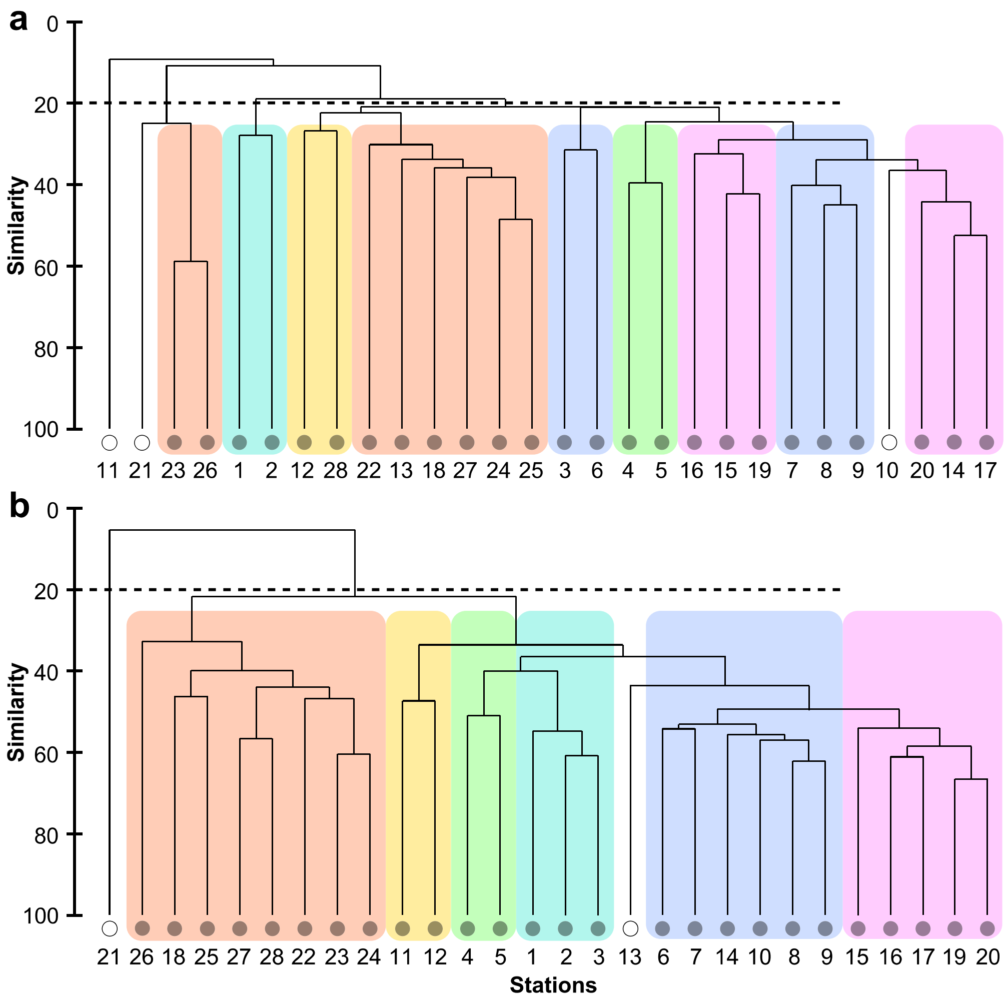

Supplementary Figure 3 CLUSTER analysis results. a The 2012 survey, conducted half year before the trawl ban. **b** The 2015 survey, conducted 2.5 years after the trawl ban. The analysis was based on fourth-root transformed species abundance data and group-average clustering. Four site groups in the 2012 survey and two site groups in the 2015 survey are clustered at 20% similarity level (dashed line). Site groups with different colour patches are significantly distinct in Bray-Curtis similarities (SIMPROF procedure, $P < 0.05$).

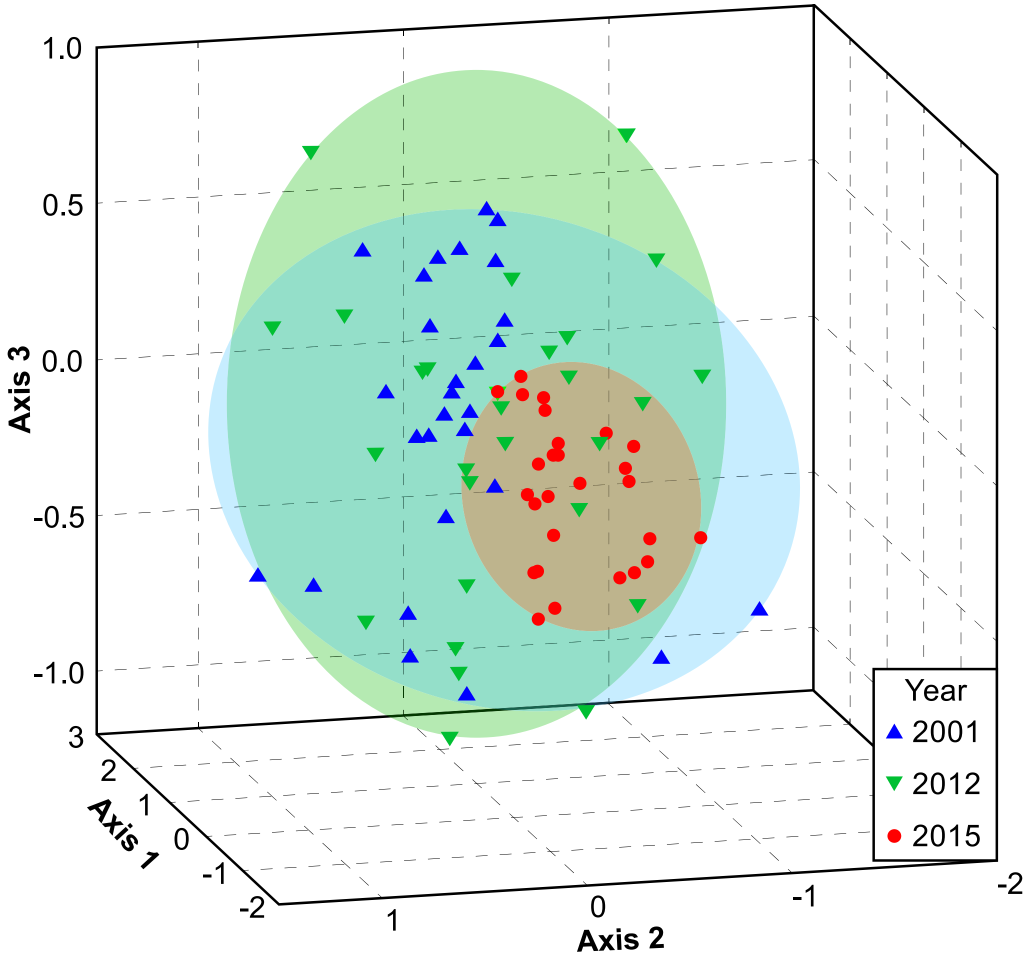

Supplementary Figure 4 Non-metric MDS configuration. The analysis was based on fourth-root transformed family abundance, covering 28 sites in the three surveys conducted in 2001 (blue), 2012 (green) and 2015 (red). The 3D stress value was 0.15.

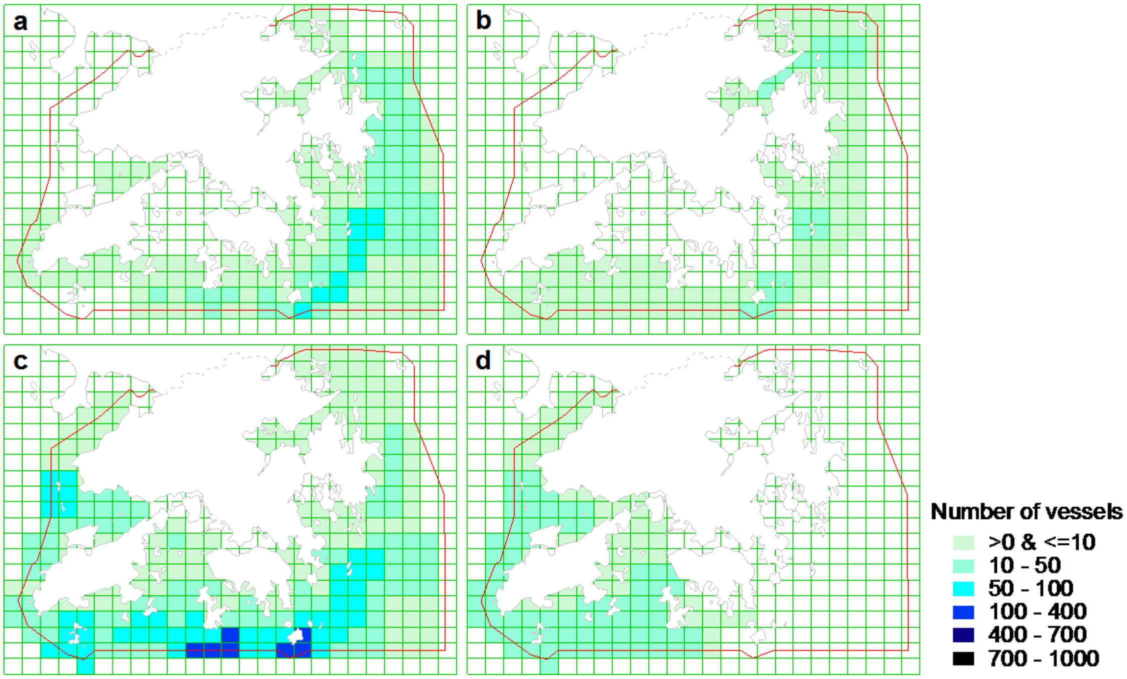

Supplementary Figure 5 Distribution of fishing operations in Hong Kong waters in 2006. a Stern trawlers. **b** Pair trawlers. **c** Shrimp trawlers. **d** Hang trawlers. Source: AFCD Port Survey 2006.

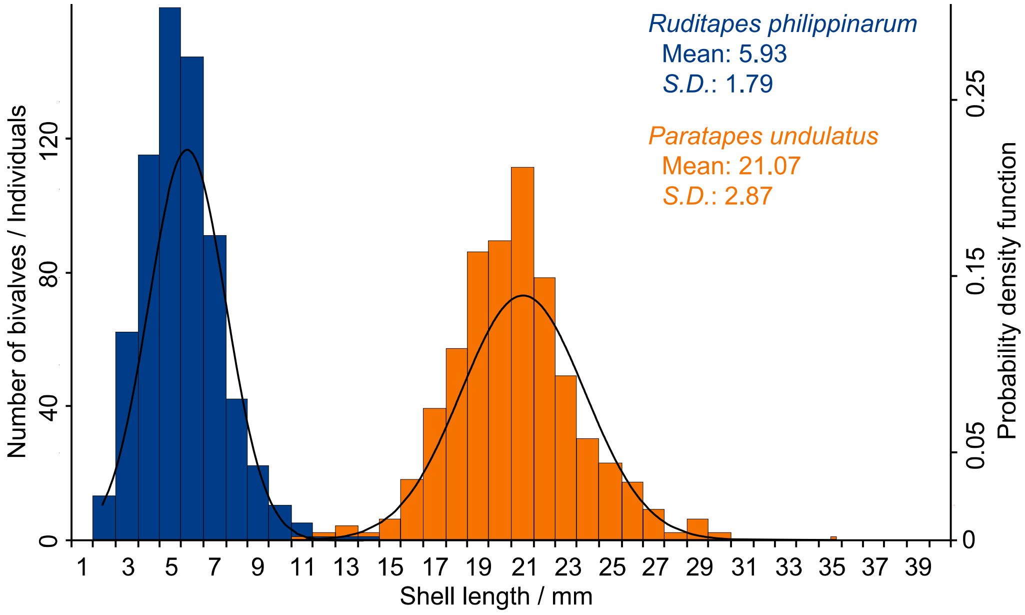

Supplementary Figure 6 Relationships between number of individuals and shell length of two bivalves after the trawl ban. The histograms in blue represents data of *Ruditapes philippinarum* collected from the Western Waters (sites 5 and 7); and the histogram in orange represents *Paratapes undulatus* collected from the Deep Bay (sites 2 and 3). Probability density curves were constructed with the function NORMDIST in software Microsoft Excel with the means and standard deviations calculated from the raw data. Neither of the two bivalves was collected from these four sites in the 2012 survey.

Supplementary Table 1 Abbreviations for abiotic variables used in this study.

Abiotic variables	Abbreviation
Chemical oxygen demand	COD
Electrochemical potential	EP
Particle size fraction < 63 μm (%)	PF_{63}
Suspended solids	SUS
Total carbon	TC
Total Kjeldahl nitrogen	TKN
Total organic matter	TOM
Total phosphorous	TP
Total sulphide	TS
Total volatile solids	TVS
Turbidity	TUR

Supplementary Table 2 Statistics for the abiotic variables between surveys.

Variables	2001		2012		*t*	*P*	Variables	2012		2015		*t*	*P*
	Mean	*S.D.*	Mean	*S.D.*				Mean	*S.D.*	Mean	*S.D.*		
COD (mg/kg)	15118	3399	12411	3501	4.562	**0.0000987*** **	COD (mg/kg)	12411	3501	14229	4034	-4.310	**0.000194*** **
EP (mV)	-205.3	94.59	-185.3	99.16	-0.982	0.335	EP (mV)	-185.3	99.16	-323.2	51	7.166	**0.000000105*** **
PF (w/w%)	81.71	19.99	75.00	21.24	1.356	0.186	PF (w/w%)	75.00	21.24	79.43	15.38	-1.358	0.186
TC (w/w%)	0.686	0.158	0.736	0.199	-1.457	0.157	TC (w/w%)	0.736	0.199	0.800	0.221	-1.819	0.0800
TKN (mg/kg)	436.1	143.0	537.1	134.8	-5.126	**0.0000217*** **	TKN (mg/kg)	537.1	134.8	577.1	121.4	-2.446	**0.0213* **
TP (mg/kg)	201.1	40.49	212.5	31.34	-1.758	0.0900	TP (mg/kg)	212.5	31.34	220.7	31.02	-1.774	0.0874
TS (mg/kg)	98.82	98.28	56.57	93.85	2.119	0.0435	TS (mg/kg)	56.57	93.85	58.06	85.35	-0.073	0.942
TOM (w/w%)	6.835	2.347	5.486	2.478	2.988	**0.00591* **	TOM (w/w%)	5.486	2.478	7.086	2.050	-6.101	**0.00000162*** **
TVS (w/w%)	7.771	1.930	7.039	1.978	2.866	**0.00796* **	TVS (w/w%)	7.039	1.978	7.729	2.169	-3.270	**0.00293** **
SUS (mg/L)	7.956	6.842	5.492	3.492	2.870	**0.00844* **	SUS (mg/L)	5.492	3.492	4.136	1.607	2.885	**0.00815* **
TUR (NTU)	13.87	7.911	4.260	2.397	8.051	**0.0000000282*** **	TUR (NTU)	4.260	2.397	3.960	1.933	1.223	0.233

Abiotic variables between surveys ($n = 28$; $n = 25$ for SUS and TUR) are compared using two-tailed paired samples *t*-test. Two statistical tests were performed for each abiotic variable, therefore only tests with $P < 0.025$ were considered significant after Bonferroni correction (Asterisks indicate significant differences, i.e., *$P < 0.025$, **$P < 0.005$, ***$P < 0.0005$). Abbreviations for abiotic variables see Supplementary Table 1.

Supplementary Table 3 Results of principal components analysis (PCA) of abiotic variables across the study area.

Variable	PC1	PC2	PC3	PC4	PC5
Chemical oxygen demand (mg/kg)	0.331	-0.500	-0.176	0.047	0.108
Electrochemical potential (mV)	-0.218	0.166	0.552	-0.161	0.632
Particle size fraction < 63µm (%)	0.231	0.313	-0.336	0.400	0.517
Total carbon (w/w%)	0.281	-0.111	0.012	-0.606	-0.142
Total Kjeldahl nitrogen (mg/kg)	0.456	0.088	-0.111	-0.033	0.088
Total phosphorous (mg/kg)	-0.008	0.471	-0.556	-0.409	0.101
Total sulphide (mg/kg)	0.233	-0.490	-0.045	-0.151	0.467
Total organic matter (w/w%)	0.366	0.090	0.171	0.471	-0.197
Suspended-solid (mg/L)	-0.396	-0.269	-0.265	0.093	0.160
Turbidity (NTU)	-0.397	-0.249	-0.359	0.153	-0.024
Eigenvalues	4.000	1.670	1.180	0.960	0.760
Cumulative % variation	40.0	56.7	68.5	78.0	85.6

Abiotic variables of 25 sites in the 2012 and 2015 surveys are used for PCA (n = 50). The results show component loadings, eigenvalues, and percentage of variance explained.

Supplementary Table 4 Comparison in biotic variables before (2012) and after (2015) the trawl ban.

Variables	**Trawled 23 sites**						**Non-trawled 5 sites**					
	2012		2015		*t*	*P*	2012		2015		*t*	*P*
	Mean	*S.D.*	Mean	*S.D.*			Mean	*S.D.*	Mean	*S.D.*		
Total abundance	189.4	375.1	672.7	853.8	-4.412	**0.000220*** **	547.6	912.9	1652	1861	-2.195	0.093
Total biomass	17.39	21.39	50.05	113.9	-1.368	0.185	286.8	626.3	166.3	352.5	0.344	0.748
Species number	24.57	13.62	46.22	24.43	-6.674	**0.00000104*** **	41.20	30.85	58.00	33.79	-1.902	0.130
Margalef's richness index, *d*	4.990	2.425	7.111	3.378	-5.089	**0.0000425*** **	6.867	4.357	8.171	4.400	-1.327	0.255
Pielou's evenness index, *J*	0.775	0.174	0.676	0.145	2.779	**0.011* **	0.768	0.064	0.678	0.122	3.422	**0.027* **
Shannon-Wiener diversity index, *H'*	3.326	1.074	3.564	1.298	-1.168	0.255	3.610	1.498	3.804	1.249	-0.548	0.613
AZTI's Marine Biotic Index, AMBI	2.593	0.834	2.305	0.836	1.265	0.219	2.227	0.579	2.250	0.521	-0.054	0.960
Functional diversity	2.458	0.711	2.445	0.807	0.093	0.926	2.484	1.084	2.509	0.649	-0.096	0.928
Functional groups	11.17	4.509	14.57	5.623	-4.177	**0.000391*** **	13.40	8.933	15.60	6.504	-1.329	0.255
Collectors	109.3	355.9	215.3	242.6	-2.292	**0.032* **	121.2	148.5	609.8	683.9	-1.926	0.126
Burrowers	16.65	17.19	72.39	98.62	-3.134	**0.005** **	179.2	353.2	74.20	95.79	0.625	0.566
Carnivores	30.22	29.41	157.2	73.80	-7.829	**0.0000000843*** **	68.00	68.73	556.2	769.4	-1.553	0.195
Surface deposit feeders	1.304	2.401	49.61	50.63	-4.573	**0.000149*** **	12.20	20.08	243.4	343.9	-1.579	0.189
Omnivores	20.83	41.70	15.78	17.55	0.691	0.497	96.60	196.7	95.40	152.5	0.057	0.958
Suspension feeders	11.09	11.27	162.4	550.9	-1.331	0.197	70.40	142.4	73.00	108.2	-0.032	0.976
Discretely motile	119.1	360.0	330.0	760.6	-2.428	**0.024* **	162.6	241.2	363.4	378.7	-1.812	0.144
Motile	65.17	57.13	320.0	227.4	-5.794	**0.00000791*** **	366.0	635.4	1269	1496	-2.115	0.102
Sessile	5.174	7.253	22.70	30.18	-3.351	**0.003** **	19.00	38.06	20.00	22.07	-0.075	0.944

Paired sample statistics and two tailed *t*-test are used for the biotic variables between the two surveys in 2012 and 2015 (Asterisks indicate significant differences, i.e., $*P < 0.05$, $**P < 0.01$, $***P < 0.001$). Trawled sites (i.e., Sites 1, 2, 3, 4, 5, 6, 7, 8, 9, 10, 15, 16, 17, 18, 19, 20, 21, 22, 23, 24, 25, 27, and 28) and un-trawled sites (i.e., Sites 11, 12, 13, 14 in the busiest shipping channel of Victoria Harbour, and Site 26 in Yan Chau Tong Marine Park) are separately displayed.

Supplementary Table 5 Feeding guilds of macrobenthos in Hong Kong waters.

Functional groups	Motile	Discretely motile	Sessile
***Suspension feeder* (S)**			
Arm spines & tube feet (A)	SMA	-	-
Choanocyte (C)	-	-	SSC
Maxillipeds (M)	SMM	-	SSM
Pumping (P)	-	SDP	-
Radula (R)	-	SDR	-
Tentacle (T)	-	-	SST
***Collector* (&)**			
Maxillipeds (M)	&MM	-	-
Pumping (P)	-	&DP	-
Tentacle (T)	&MT	&DT	&ST
Unarmed pharynx (U)	-	&DU	-
***Surface deposit feeder* (D)**			
Jawed pharynx (J)	DMJ	-	-
Maxillipeds (M)	DMM	-	-
Tentacle (T)	-	-	DST
Teeth (E)	DME	-	-
Unarmed pharynx (U)	DMU	-	-
***Burrower* (B)**			
Maxillipeds (M)	BMM	-	-
Radula (R)	BMR	-	-
Tentacle (T)	BMT	BDT	-
Unarmed pharynx (U)	BMU	-	BSU
***Carnivore* (C)**			
Jawed pharynx (J)	CMJ	CDJ	-
Maxillipeds (M)	CMM	-	-
Radula (R)	-	CDR	-
Unarmed pharynx (U)	CMU	-	-
***Omnivore* (O)**			
Jawed pharynx (J)	OMJ	ODJ	-
Maxillipeds (M)	OMM	ODM	-
Radula (R)	-	ODR	-
Unarmed pharynx (U)	OMU	-	-

First letter code: major feeding mode; Second letter code: motility pattern; Third letter code: morphological structure used in feeding. -: No macrobenthos fit for such functional group.
