# Supplementary material for: Recovery of tropical marine benthos after a trawl ban demonstrates linkage between abiotic and biotic changes
Source: Commun Biol. 2021 Feb 16;4:212. doi: 10.1038/s42003-021-01732-y (PMC7887210; doi:10.1038/s42003-021-01732-y)
Supplement: Supplementary file 3 — Description of Additional Supplementary Files [file 42003_2021_1732_MOESM3_ESM.pdf]

Description of Additional Supplementary Files

Supplementary Data 1: Source data underlying the graphs and charts presented in the main figures.

The sheet “Fig. 1c_SUS” contains the suspended solids data in each site between years used in making the “SUS” chart in Fig. 1. The sheet “Fig. 1c_COD,TKN,TOM” contains COD, TKN and TOM data in each site between years used in making the “COD”, “TKN” and “TOM” chart in Fig. 1. The sheet “Fig. 2a,b,c,d” contains the total abundance/(inds./0.5m^2), total biomass/(g/0.5m^2), Margalef’s richness index (*d*), functional groups data in each site between years used for the charts of Fig. 2a, b, c, d. The sheet “Fig.2e,f” contains the Bray-Curtis similarity matrix data between years in 2012 and 2015 used for making the NMDS plots of Fig.2e, f. The sheet “Fig. 3_CAP” contains the Bray-Curtis similarity matrix data in 2001(a1-a28), 2012(b1-b28), 2015(c1-c28) used for making the CAP graph in Fig. 3.

Supplementary Data 2: Taxonomic groups, feeding guilds and ecological groups of macrobenthos in the surveyed sites in Hong Kong waters.
