# Supplementary material for: Recovery of tropical marine benthos after a trawl ban demonstrates linkage between abiotic and biotic changes
Source: Commun Biol. 2021 Feb 16;4:212. doi: 10.1038/s42003-021-01732-y (PMC7887210; doi:10.1038/s42003-021-01732-y)
Supplement: Supplementary file 6 — Reporting Summary [file 42003_2021_1732_MOESM6_ESM.pdf]

nature research

Corresponding author(s): Kenneth Leung

Last updated by author(s): Jan 11, 2021

Reporting Summary

Nature Research wishes to improve the reproducibility of the work that we publish. This form provides structure for consistency and transparency in reporting. For further information on Nature Research policies, see our Editorial Policies and the Editorial Policy Checklist.

Statistics

For all statistical analyses, confirm that the following items are present in the figure legend, table legend, main text, or Methods section.

n/a	Confirmed	
☐	☒	The exact sample size (*n*) for each experimental group/condition, given as a discrete number and unit of measurement
☐	☒	A statement on whether measurements were taken from distinct samples or whether the same sample was measured repeatedly
☐	☒	The statistical test(s) used AND whether they are one- or two-sided<br>*Only common tests should be described solely by name; describe more complex techniques in the Methods section.*
☒	☐	A description of all covariates tested
☐	☒	A description of any assumptions or corrections, such as tests of normality and adjustment for multiple comparisons
☐	☒	A full description of the statistical parameters including central tendency (e.g. means) or other basic estimates (e.g. regression coefficient) AND variation (e.g. standard deviation) or associated estimates of uncertainty (e.g. confidence intervals)
☐	☒	For null hypothesis testing, the test statistic (e.g. *F*, *t*, *r*) with confidence intervals, effect sizes, degrees of freedom and *P* value noted<br>*Give P values as exact values whenever suitable.*
☒	☐	For Bayesian analysis, information on the choice of priors and Markov chain Monte Carlo settings
☐	☒	For hierarchical and complex designs, identification of the appropriate level for tests and full reporting of outcomes
☐	☒	Estimates of effect sizes (e.g. Cohen's *d*, Pearson's *r*), indicating how they were calculated

Our web collection on statistics for biologists contains articles on many of the points above.

Software and code

Policy information about availability of computer code

Data collection	No software was used in Data collection.
Data analysis	1) SPSS Statistics v.17.0 software for performing two-tailed Pearson correlation test, paired samples t-test, and independent samples t-test among abiotic and biotic variables; 2) "FactoMineR" package in the R-Studio 1.0.143 for performing PCA, and "CAR" package for detecting multi-collinearity; 3) "CLUSTER" procedure in the PRIMER 6 software for determining the spatial and temporal variations in community structure, "SIMPROF" procedure for detecting significantly clustered site groups, "Non-metric multidimensional scaling (NMDS)" procedure for constructing 3D configurations of sites, "PERMANOVA" procedure were used to compare the Bray-Curtis similarities between surveys, and "Canonical Analysis of Principal Coordinates (CAP)" procedure was further used to plot the ordination of sites in different surveys.

For manuscripts utilizing custom algorithms or software that are central to the research but not yet described in published literature, software must be made available to editors and reviewers. We strongly encourage code deposition in a community repository (e.g. GitHub). See the Nature Research guidelines for submitting code & software for further information.

Data

Policy information about availability of data

All manuscripts must include a data availability statement. This statement should provide the following information, where applicable:

- Accession codes, unique identifiers, or web links for publicly available datasets
- A list of figures that have associated raw data
- A description of any restrictions on data availability

All datasets support the findings of this study are available from the corresponding author upon request.

Field-specific reporting

Please select the one below that is the best fit for your research. If you are not sure, read the appropriate sections before making your selection.

☐ Life sciences ☐ Behavioural & social sciences ☒ Ecological, evolutionary & environmental sciences

For a reference copy of the document with all sections, see nature.com/documents/nr-reporting-summary-flat.pdf

Ecological, evolutionary & environmental sciences study design

All studies must disclose on these points even when the disclosure is negative.

Study description	This study was conducted to collect macrobenthic samples from 28 sites in the tropical Hong Kong waters in June 2012 and June 2015. The 28 survey sites located in three hydrological regions (i.e., Estuarine, Transitional and Oceanic) and seven Water Control Zones in Hong Kong. At each site, six sediment samples were collected, five for biotic analyses and one for abiotic analyses.
Research sample	This study collected a total of 429 macrobenthos species (123 families in 8 phyla in the 2012 survey, and 119 families in 8 phyla in the 2015 survey), inlcuding Polychaetes (e.g., Oxydromus obscurus), Molluscs (e.g., Paratapes undulatus), Crustacean (e.g., Typhlocarcinus villosus), and echinoderms (e.g., Acaudina sp.), and other invertebrates.
Sampling strategy	Five sediment samples for faunal analysis and one sediment sample for sediment analysis were collected using a 0.1 m2 van Veen grab at each site.
Data collection	In the laboratory, macrobenthic samples were rinsed with freshwater (by Yanan Sun, Yang James Xie, Yanjie Zhang, Ting Xu and Jian-Wen Qiu), then picked up from the sieved residues and transferred to 70% ethanol (by Yanan Sun, Yang James Xie, Yanjie Zhang, Ting Xu and Zhi Wang) and later identified to the lowest possible taxonomic level (by Institute of Oceanology Chinese Academy of Sciences, Yanan Sun and Zhi Wang). Abundance was determined by counting only specimens with anterior fragment (by Institute of Oceanology Chinese Academy of Sciences, Yanan Sun and Zhi Wang). Samples were then blotted dry with a paper towel and weighed using an electronic balance (by Institute of Oceanology Chinese Academy of Sciences, Yanan Sun and Zhi Wang).
Timing and spatial scale	Samples were collected on 5-8 June 2012 and on 8, 9, 17, 29, 30 June 2015.
Data exclusions	Before the PCA procedure, multi-collinearity among the abiotic variables was detected with the VIF (variance inflation factor) command in the “CAR” package, and variables (here refers to total volatile solids) had a clear sign of collinearity (i.e., VIF value > 10) were excluded from further analysis.
Reproducibility	Further studies based on collection of faunal and sediment samples in the surveyed sampling sites in sub-tidal Hong Kong waters could be useful to verify the reproducibility of our experimental findings.
Randomization	The Canonical Analysis of Principal Coordinates (CAP) in this study aim to compare differences among three surveys in 2001, 2012, and 2015. So abundance data of macrobenthos in the 28 surveyed sites were allocated in groups according to years before the CAP procedure.
Blinding	Blinding was not relevant to this study.

Did the study involve field work? ☒ Yes ☐ No

Field work, collection and transport

Field conditions	Field sampling work was conducted in the tropical Hong Kong waters in June 2012 and June 2015. Total Precipitation was 261.5mm in June 2012 and 302.1 mm in June 2015. Average temperature 26.0-29.9 Celsius degree in June 2012 and 27.3-30.9 Celsius degree in June 2015.خ
Location	Sampling was taken in the Hong Kong waters (113° 53' 29.6959"E-114° 25' 53.3783"E, 22° 09' 11.7445"N-22° 33' 35.5757"N), water depth from 1.1m-47.9m.
Access & import/export	Not applicable.
Disturbance	For the abiotic variables, natural and anthropogenic disturbances might have influenced estimation of the SUS and turbidity loads. To minimize the random effects, mean values of SUS and turbidity data from 12 months before and on the sampling month (July 2000-June 2001, July 2011-June 2012, July 2014-June 2015; measurement once per month) of the surveyed 25 sites were used in subsequent data analyses.

Reporting for specific materials, systems and methods

We require information from authors about some types of materials, experimental systems and methods used in many studies. Here, indicate whether each material, system or method listed is relevant to your study. If you are not sure if a list item applies to your research, read the appropriate section before selecting a response.

Materials & experimental systems

n/a	Involved in the study
☒	☐ Antibodies
☒	☐ Eukaryotic cell lines
☒	☐ Palaeontology and archaeology
☐	☒ Animals and other organisms
☒	☐ Human research participants
☒	☐ Clinical data
☒	☐ Dual use research of concern

Methods

n/a	Involved in the study
☒	☐ ChIP-seq
☒	☐ Flow cytometry
☒	☐ MRI-based neuroimaging

Animals and other organisms

Policy information about studies involving animals; ARRIVE guidelines recommended for reporting animal research

Laboratory animals	Not applicable.
Wild animals	Not applicable.
Field-collected samples	Five sediment samples for faunal analysis and one sediment sample for sediment analysis were collected using a 0.1 m2 van Veen grab at each site. The faunal samples were gently rinsed through a 0.5 mm-mesh sieve at sea. Residues retained on the sieve, including macrobenthos, were transferred into labelled plastic bags, fixed in 5% formalin solution in seawater and stained with 1% Rose Bengal. Approximately 400 g sediment at each site were scooped into a plastic bag for sediment analysis, kept on ice on board in a cooler, and transported to the laboratory and frozen at -20 °C in a freezer.
Ethics oversight	No ethical approval or guidance was required. This study basically focused on the collection, identification and analysis of marine invertebrates and there was no violation of ethics during the study.

Note that full information on the approval of the study protocol must also be provided in the manuscript.
